# Supplementary figures and images for: Puzzle Imaging: Using Large-Scale Dimensionality Reduction Algorithms for Localization
Source: PLoS One. 2015 Jul 20;10(7):e0131593. doi: 10.1371/journal.pone.0131593 (PMC4507868; doi:10.1371/journal.pone.0131593)

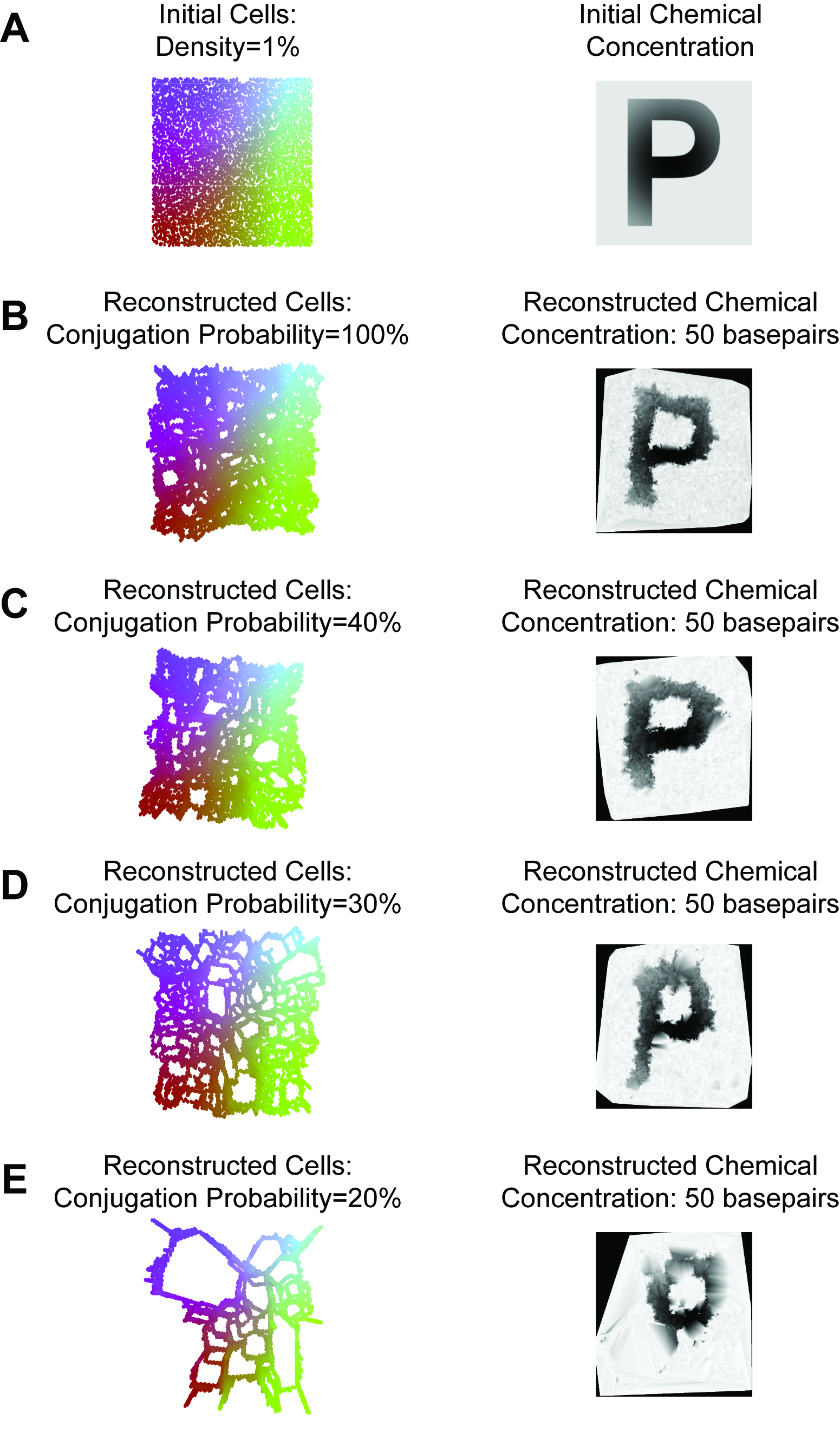

Supplement: S1 Fig — We do a simulation of chemical puzzling, as in Fig 7B, except now with varying conjugation efficiencies. (A) The original cell locations (left), and initial chemical concentration (right). (B-E) On the left, the reconstructed cells, and on the right, the reconstructed chemical concentration, using (B) 100% conjugation probability, (C) 40% conjugation probability, (D), 30% conjugation probability, and (E) 20% conjugation probability. The reconstructed chemical concentrations assumed the pioneer cells had 50 base pairs to encode the concentration. The black area on the outside is a border, not a chemical concentration. (TIF) [file pone.0131593.s001.tif]
