# Supplementary material for: Puzzle Imaging: Using Large-Scale Dimensionality Reduction Algorithms for Localization
Source: PLoS One. 2015 Jul 20;10(7):e0131593. doi: 10.1371/journal.pone.0131593 (PMC4507868; doi:10.1371/journal.pone.0131593)
Supplement: S1 Text — A discussion about our assumptions regarding conjugation efficiency in our Chemical Puzzling simulations, and results about what happens when those assumptions are relaxed. (PDF) [file pone.0131593.s003.pdf]

Chemical Puzzling Conjugation Efficiency

The simulations in the main text (Fig. 7) assumed that when an F^+ and F^- cell are in contact, transfer of genetic information occurs 100% of the time. Evidence to the contrary exists, especially in the absence of selection, where the fitness penalty associated with F^+ strains carrying a plasmid, and the competition between cell growth and conjugation, limit plasmid transfer [1]. Furthermore, recombination of the F-plasmid with the host genome can result in Hfr (high frequency of recombination) strains, which have the ability to transfer genetic information (e.g. barcodes), but generally do not confer conjugative ability to the recipient [2]. Our analysis, however, does not rely on every neighboring F^+/F^- pair to mate. Rather, it only requires that the descendants of nearby pioneer cells mate at least one once (if they are an F^+/F^- pair). In simulations, relatively faithful chemical reconstruction was accomplished with a conjugation probability of only 30% (Fig. S1). This probability is likely very feasible, as neighboring pairs will have many chances to mate.

References

[1] Ponciano JM, De Gelder L, Top EM, Joyce P. The population biology of bacterial plasmids: a hidden Markov model approach. Genetics. 2007;176(2):957–968.

[2] Gupta P. Cell and Molecular biology. Rastogi Publications; 2009.
