# Supplementary material for: Puzzle Imaging: Using Large-Scale Dimensionality Reduction Algorithms for Localization
Source: PLoS One. 2015 Jul 20;10(7):e0131593. doi: 10.1371/journal.pone.0131593 (PMC4507868; doi:10.1371/journal.pone.0131593)
Supplement: S2 Text — Further discussion on the limitations of our simulations. (PDF) [file pone.0131593.s004.pdf]

Simulation Limitations

Neural Voxel Puzzling

In our simulations, we aimed to demonstrate some of the problems that could arise when using real data. For instance, for voxel puzzling, we removed a large portion of the voxels to represent cell bodies taking up multiple voxels. It would likely be possible to place these voxels when using real data, by looking at the surrounding voxels that share a neuron tag.

In our voxel puzzling simulations, there were equal densities of neurons in all portions of the volume, which is not the case in the brain. This should not be a large concern, however, due to our method of constructing the similarity matrix. When we do thresholding, we are essentially taking the approximate nearest neighbors. This step removes the effects of having more total connections between voxels in one region (due to a high density of neurons) compared to another.

Our neurons were also all oriented at random angles, so that voxels that were horizontal of each other had the same probability of sharing a neuron as voxels that were vertical of each other (or on top of each other). If this is not the case (e.g. axons are oriented in a similar manner in many brain regions), the reconstruction will be skewed. For instance, if most neurons were oriented vertically, in the reconstruction, the vertical component will be compressed. Following reconstruction, if it is seen that many neurons in a region are all oriented in the same manner, the dimensions of this region could be scaled proportionally. Moreover, having molecular annotations about whether axons or dendrites of a neuron are in a voxel could help with this process.

Neural Connectomics Puzzling

In our simulations of neural connectomics puzzling, we demonstrated that accurate reconstructions were still possible when there were multiple connection probability distributions (as a function of distance). However, this simulation only had 2 layers, and the connection probability distributions were consistent in each layer. It is possible that reconstructions could be less accurate when using more layers. This could be dealt with by using *a priori* knowledge about neuroanatomy in order to ensure cubes were cut to contain a homogenous population. It also could be problematic if connection probability distributions differed within a layer across multiple cell types [1]. This could be dealt with by using annotated molecular information about cell types [2]. In this scenario, separate reconstructions could be done using neuronal populations with homogenous connectivity probability functions, and then these reconstructions could be combined. Additionally, it may be possible to estimate cell types given connectivity information (by using clustering methods on the connectivity data) [3], and then use these cell type estimates (as mentioned above) to help localize the neurons.

It is also important to note that connectomics puzzling is based on the idea that there is a higher probability of connection between neurons that are closer to each other. If this is not the case, then the connectomics puzzling reconstruction may be inaccurate. For certain cell types or portions of the brain (e.g. layer 5 pyramidal cells), there may be a higher probability of long-range connections than short connections. Using *a priori* knowledge about neuroanatomy or information about cell types based on molecular annotations, these neurons could be excluded (or specific connections these neurons have could be excluded). Alternatively, this information about long-range connections could be utilized in connectomics puzzling to help provide an accurate reconstruction.

Chemical Puzzling

In our simulations of chemical puzzling, we used simplified models of bacterial growth and movement. In terms of cell growth, we assumed that cell colonies keep growing outward and stop once they contact another colony. In reality, cells will push on one another, causing the colonies to move. Additionally, in our two

dimensional simulations, we ignored the possibility of cells growing in the vertical dimension, which could lead to additional conjugations. While we could incorporate a more sophisticated (and much more expensive in terms of CPU time) simulation of bacterial growth, it is important to note that these simplifications will have little effect on whether two colonies have a conjugation, which is the metric that influences our results.

Our simulations also assumed that cells are stationary; that is, once a cell occupies a particular spot in two (or three) dimensional space, it stays there forever. In this case, the trajectory of a lineage is determined by a two (or three) dimensional random walk with uniform and unity step size, where two trajectories do not cross. This would certainly not be true in the case of motile cells or when there is sufficient mixing, as cells arising from different lineages could cross before dividing. Further, if the particular chemical being sensed induces a bias in cell motility (i.e. is a chemoattractant or chemorepellant), a bias will be further introduced into the colony growth model. These movement affects could potentially be problematic for two reasons: 1) it could allow far-away colonies to conjugate with one another; 2) pioneer cells could determine the chemical concentration of a location different from the location where they start to divide. Detailed analysis of these movement affects is beyond the scope of this paper, and we simply note that in the case that cell motility is also an unbiased random walk (a good assumption in the absence of chemotaxis and viscous flow [4]) and there is no mixing, our analysis stands in the limit that the average distance covered by the cells during one division time is much smaller than their size. Outside of this limit, our reconstructed images would be blurred.

References

[1] Liley D, Wright J. Intracortical connectivity of pyramidal and stellate cells: estimates of synaptic densities and coupling symmetry. Network: Computation in Neural Systems. 1994;5(2):175–189.

[2] Usoskin D, Furlan A, Islam S, Abdo H, Lnnerberg P, Lou D, et al. Unbiased classification of sensory neuron types by large-scale single-cell RNA sequencing. Nature neuroscience. 2014.

[3] Jonas E, Kording K. Automatic discovery of cell types and microcircuitry from neural connectomics. eLife. 2015;4:e04250.

[4] Saragosti J, Silberzan P, Buguin A. Modeling E. coli tumbles by rotational diffusion. Implications for chemotaxis. PloS one. 2012;7(4):e35412.
